# Potential Anticancer Activity of *Juniperus procera* and Molecular Docking Models of Active Proteins in Cancer Cells

**DOI:** 10.3390/molecules28052041

**Published:** 2023-02-22

**Authors:** Sultan Alhayyani, Abdullah Akhdhar, Amer H. Asseri, Abdelhafeez M. A. Mohammed, Mostafa A. Hussien, L. Selva Roselin, Salman Hosawi, Fahad AlAbbasi, Khadijah H. Alharbi, Roua S. Baty, Abdulaziz A. Kalantan, Ehab M. M. Ali

**Affiliations:** 1 Department of Chemistry, College of Sciences & Arts, King Abdulaziz University, Rabigh 21911, Saudi Arabia; 2 Department of Chemistry, College of Science, University of Jeddah, Jeddah 21589, Saudi Arabia; 3 Biochemistry Department, Faculty of Science, King Abdulaziz University, Jeddah 21589, Saudi Arabia; 4 Centre for Artificial Intelligence in Precision Medicines, King Abdulaziz University, Jeddah 21589, Saudi Arabia; 5 Department of Chemistry, Faculty of Education, Alzaiem Alazhari University, Khartoum 13311, Sudan; 6 Department of Chemistry, Faculty of Science, King Abdulaziz University, Jeddah 21589, Saudi Arabia; 7 Department of Chemistry, Faculty of Science, Port Said University, Port Said 42521, Egypt; 8 Department of Biotechnology, College of Science, Taif University, Taif 21944, Saudi Arabia; 9 Division of Biochemistry, Chemistry Department, Faculty of Science Tanta University, Tanta 31527, Egypt

**Keywords:** *Juniperus procera*, anticancer, cytotoxicity, apoptosis, cell cycle, GC/MS analysis, molecular docking, Cdk5

## Abstract

Medicinal plants provide a wide range of active compounds that can be exploited to create novel medicines with minimal side effects. The current study aimed to identify the anticancer properties of *Juniperus procera* (*J. procera*) leaves. Here, we demonstrate that *J. procera* leaves’ methanolic extract suppresses cancer cells in colon (HCT_116_), liver (HepG2), breast (MCF-7), and erythroid (JK-1) cell lines. By applying GC/MS, we were able to determine the components of the *J. procera* extract that might contribute to cytotoxicity. Molecular docking modules were created that used active components against cyclin-dependent kinase 5 (Cdk5) in colon cancer, aromatase cytochrome P450 in the breast cancer receptor protein, the -N terminal domain in the erythroid cancer receptor of the erythroid spectrin, and topoisomerase in liver cancer. The results demonstrate that, out of the 12 bioactive compounds generated by GC/MS analysis, the active ingredient 2-imino-6-nitro-2*H*-1-benzopyran-3-carbothiamide proved to be the best-docked chemical with the chosen proteins impacted by DNA conformational changes, cell membrane integrity, and proliferation in molecular docking studies. Notably, we uncovered the capacity of *J. procera* to induce apoptosis and inhibit cell growth in the HCT_116_ cell line. Collectively, our data propose that *J. procera* leaves’ methanolic extract has an anticancer role with the potential to guide future mechanistic studies.

## 1. Introduction

Cancer is a condition that can start in any organ or tissue of the body and then spreads to other organs due to the uncontrolled growth of cells. It is the second-most common cause of death, just after heart disease. By 2030, it is anticipated that there will be 26 million cancer patients [1]. Malignant tumors can be treated surgically, with chemotherapy, and at various stages of development with radiotherapy. Myelotoxicity, cardiotoxicity, renal toxicity, pulmonary toxicity, skin toxicity, intestinal damage, and hair loss are just a few of the negative effects of chemotherapy, which can harm healthy cells [2,3]. Globally, intensive research is being conducted to create a medication that is effective at specifically destroying cancer cells. Plant extracts are not poisonous in large quantities. Plant extracts are typically mixed with chemotherapy medications to lessen the dosage and the side effects of the latter [4]. Medicinal plants’ active ingredients can be used to treat various illnesses and as adjuvants to reduce the side effects of chemotherapy [5,6].

A wide variety of plants and animals characterize the Kingdom of Saudi Arabia. One of Saudi Arabia’s most valued medicinal plants is *Juniperus procera* Hochst. ex Endl. (*Cupressaceae*), also known as Arar in Arabic and as African pencil cedar in English. *J. procera* has a pleasant scent since it contains a significant amount of volatile oils [7,8]. The *Cupressaceae* plant family contains 70 different plant species, including the juniper plant. *J. procera* is found in Saudi Arabia, Yemen, Sudan, Eritrea, and Ethiopia, as well as in Eastern Africa, from the Eastern Democratic Republic of the Congo all the way to Malawi and Zimbabwe. It is also grown in South Africa, France, the United Kingdom, the United States, India, and Australia, among other places [7,9,10]. *Juniperus* species have traditionally been used to treat hypoglycemia [11], anti-inflammatory disorders [12], cancer [13], tuberculosis, bronchitis, pneumonia, ulcers, intestinal worms, wounds, and liver disease [7].

The essential oils of *J. procera* Hochst. ex Endl. leaves and stems, as well as its ripe and unripe fruits, were evaluated for pinene, -3-carene [14]. The essential oil of *J. procera* is larvicidal against *Anopheles arabiensis*, *Patton instar* larvae, the primary malaria vector [15], and insecticidal against *Aedes aegypti* [16].

Plant bioactive substances in the diet may be helpful as cancer-prevention agents. Bioactive compounds have been proposed to suppress cancer-cell development through two mechanisms: the alteration of redox status and interference with basic cellular functions, such as apoptosis, cell cycle, angiogenesis, invasion, and metastasis [17]. Plants are used to make four types of anticancer drugs: camptothecin derivatives (camptotecin and irinotecan), taxanes (paclitaxel and docetaxel), vinca alkaloids (vinblastine, vincristine, and vindesine), and epipodophyllotoxins (etoposide and teniposide) [18]. It is feasible to develop even more potent medications from various plants. Numerous plants with anticancer properties have been examined to determine how medicinal plants cause cell-cycle arrest and prevent angiogenesis in tumor cells, in addition to inducing apoptosis and inhibiting invasiveness and metastasis [19]. The cytotoxic effect of *J. procera* leaves and fruit extracts with silver nanoparticles was investigated against the colon cancer (Caco2) cell line. The fruit extract was more effective than the leaves, and combining AgNPs with leaves or fruits demonstrated considerable anticancer efficacy [20]. *Juniperus communis* extract is also being studied to prevent melanoma carcinogenesis as it inhibits tumor development and promotes apoptosis. The inhibitory action of biochemicals on cancer cells via apoptosis is thought to be an excellent mechanism for optimum anticancer medications since apoptosis can remove damaged cells without generating inflammation [21].

The present study demonstrates, for the first time, the possible anticancer activity of the methanolic extract of *J. procera* leaves against human cell lines from the colon (HCT_116_), liver (HepG2), breast (MCF-7), and erythroid (JK-1) tissues through apoptosis and antiproliferation. The bioactive elements in the leaf extract from *J. procera* were also shown to interact with proteins involved in conformational changes in DNA, cell membrane integrity, and proliferation via molecular docking studies.

## 2. Results

### 2.1. Extraction of Bioactive Compounds

From 50 g of dried, crude, powdered leaves of *J. procera*, the methanol-extracted *J. procera* yielded the most (2.2 g) bioactive compounds, followed by *n*-hexane (1.5 g), ethyl acetate (1.1 g), and dichloromethane (0.9 g).

### 2.2. Effect of Methanolic Extract of J. procera on the Proliferation of Several Cancer Cell Lines

**Figure 1:**
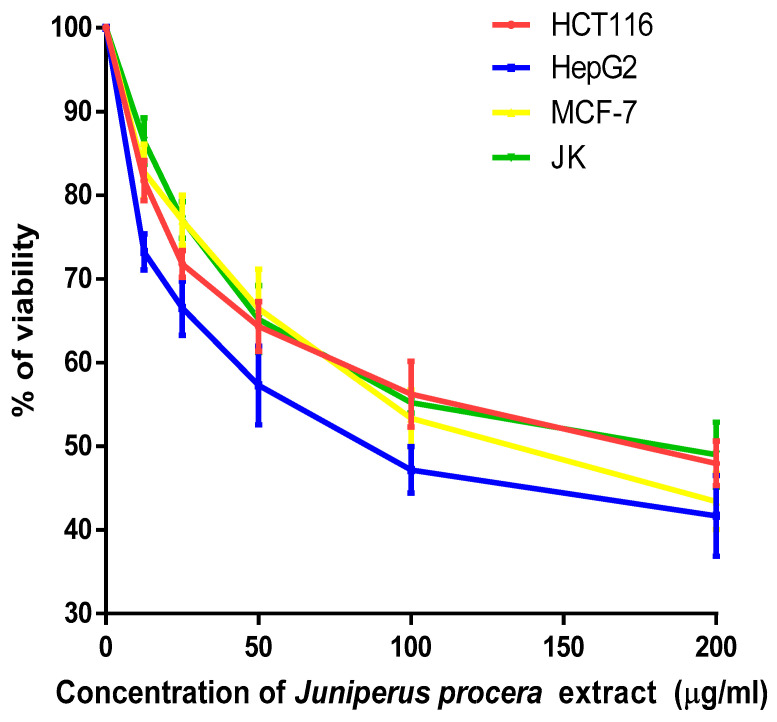
The percentage of cell viability at various concentrations of *J. procera* methanolic extract after 48 h of treatment.

The percentage viability of HCT_116_, HepG2, MCF-7, and JK-1 cells after 48 h of treatment with different dosages of *J. procera* methanolic extract is shown in Figure 1. *J. procera* extract cytotoxicity was assessed in terms of IC_50_ values for each cell line, which were 115, 75, 112, and 124 μg/mL in HCT_116_, HepG2, MCF-7, and JK-1 cells, respectively (Table 1).

### 2.3. GC/MS Analysis of Juniperus procera Leaf Extract and Molecular Docking Study

**Figure 2:**
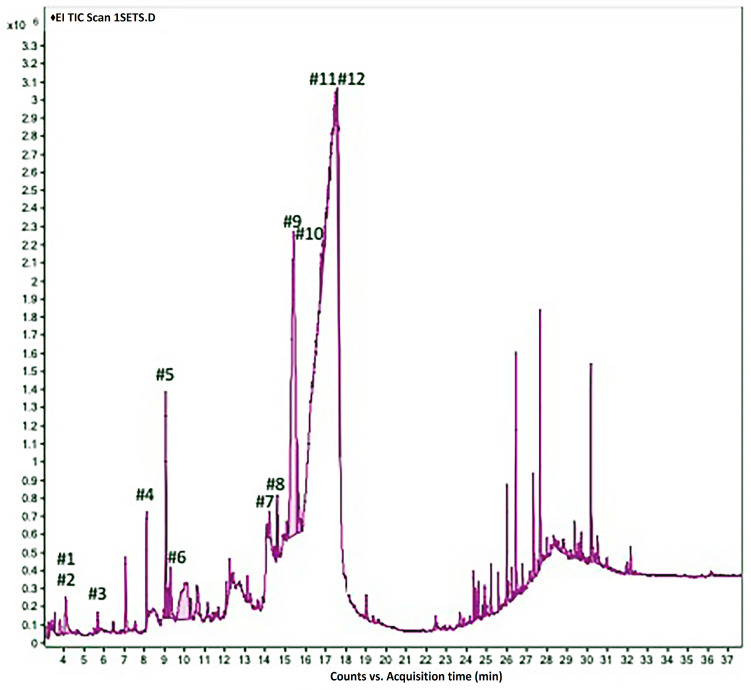
GC/MS chromatogram of *J. procera* methanol leaf extract.

**Figure 3:**
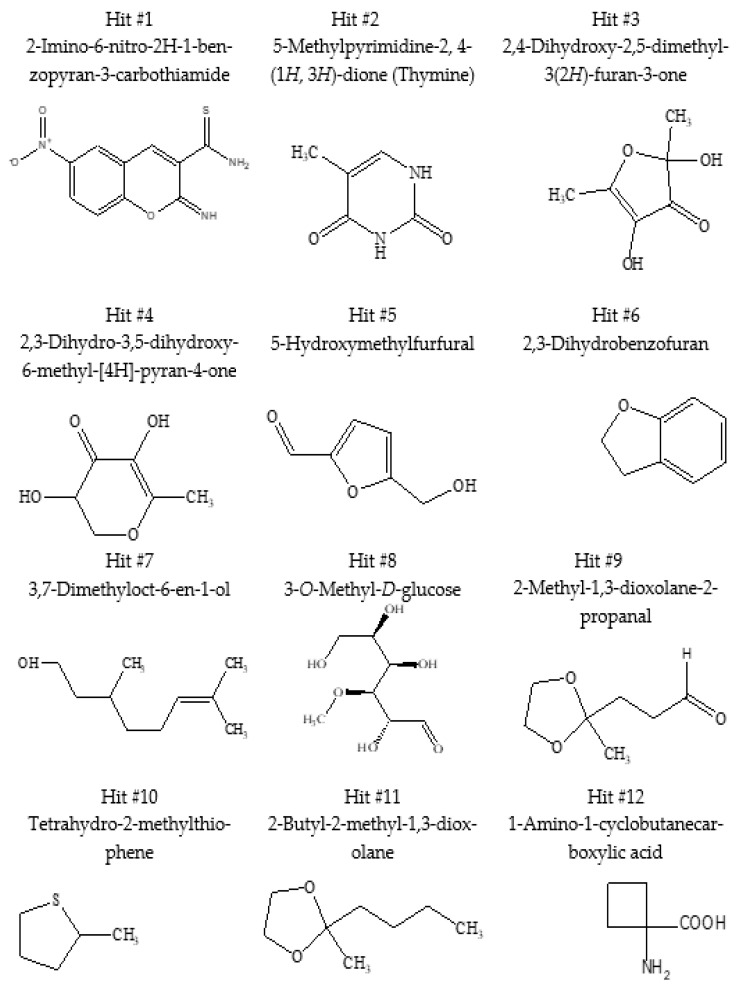
The chemical structures of bioactive compounds extracted from *J. procera* leaves and used in molecular docking experiments.

GC/MS analysis was used to determine the phytochemical contents of *J. procera* leaf extract. The chromatogram in Figure 2 shows that 12 distinct chemicals were found. The retention time and mass spectra of the reference substances in software libraries were compared to identify each peak. The compounds’ identification and structure were determined by comparing the retention time (RT) and fragmentation pattern in mass spectra to the NIST library database. Table 2 displays the chemical name, molecular formula, and retention time in the chromatogram of bioactive components found in *J. procera* extract. The molecular structure of these bioactive chemicals is depicted in Figure 3. These substances are classified as alkaloids, terpenoids, polyphenols, glycosides, flavonoids, and amino acids.

The GC/MS analysis confirmed the presence of 12 distinct active components in the methanolic extract of *J. procera* leaves (Table 2 and Figure 3). These substances were utilized in molecular docking experiments.

Table 3 displays the docking score (S), the root-mean-square deviation (RMSD), and the energy values (E) acquired during the docking analysis. The S results show that, the more negative the docking score, the better the docking between bioactive chemicals and all various proteins. Furthermore, lower RMSD values indicate a more stable docking complex. The energy values are related to the energy required for bioactive chemical binding to all proteins. As a result, less energy is required for binding, resulting in easier interaction between bioactive chemicals and proteins. The redocking data indicated that ligands were coupled to their targets in very near proximity to their real conformation, confirming the dependability of the docking techniques and settings.

According to the docking data, the chemical 2-imino-6-nitro-2*H*-1-benzopyran-3-carbothiamide (Hit 1 or Compd. **1**) is best docked with all of the distinct proteins of 3ig7, 1woa, 3eqm, and 4fm9, with docking scores of -5.56-, -5.88-, -671-, and -5.66-, respectively. The best docking score of Hit 1 was achieved against 3eqm, representing erythroid cancer protein, which matches the experimental findings.

**Figure 4:**
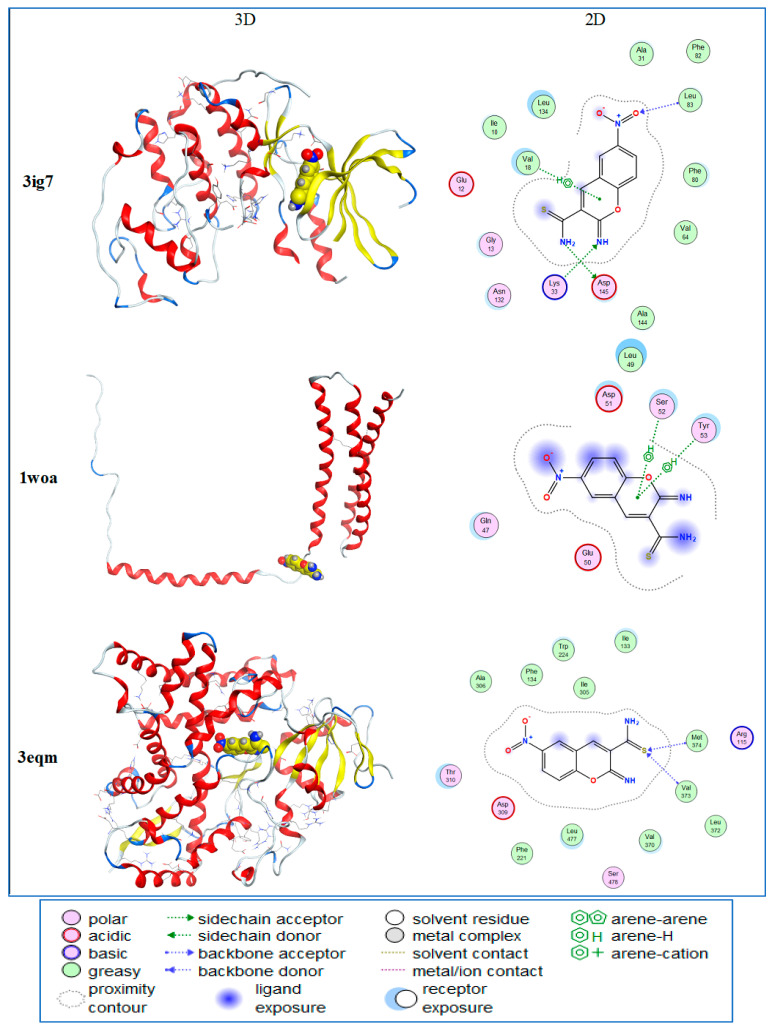
Molecular docking of 2*H*-1-benzopyran-3-carbothiamide with the receptors cyclin-dependent kinase 5 (3ig7), erythroid spectrin (1woa), aromatase cytochrome P450 (3eqm), and topoisomerase (4fm9).

Figure 4 depicts the molecular docking of 2*H*-1-benzopyran-3-carbothiamide (Hit 1) with 3ig7 CDK-5 (receptor). The CDK-5/Hit 1 complex was found in the residues Leu83, Lys33, Asp145, and Val18. The Hit 1 compound (ligand) interacts with Lys33 and Asp145 in CDK-5 (receptor) through a hydrogen bond. Lys33 is identified as an H-donor to the -NH group, whereas Asp145 is an H-acceptor from the -NH_2_ group. A hydrophobic interaction between Val18 and the aromatic ring of the Hit 1 chemical was found. The Hit 1 chemical interacts with Leu83 through a backbone interaction via an electron-withdrawing nitro group. In the instance of the erythroid spectrin/Hit 1 (1woa/Hit 1) complex, the residues contribute the most to Ser52 and Tyr53. The interaction of Ser52 and Tyr53 with the aromatic ring of the Hit 1 chemical was recognized as a hydrophobic interaction.

The aromatase cytochrome P450/Hit 1 (3eqm/Hit 1) complex in the residues was mostly responsible for Val373 and Met374. The Hit 1 compound (ligand) interaction with Val373 and 3eqm (receptor) were found to be an H-donor to the -C=S group.

In contrast, the topoisomerase/Hit 1 (4fm9/Hit 1) complex in the residues contributes significantly to Arg673. The Hit 1 compound (ligand) interactions with Arg673 and 4fm9 (receptor) were discovered to act as an H-donor to the -C=S group.

### 2.4. Flow-Cytometry Assessment of Apoptosis and Cell-Cycle Analysis

**Figure 5:**
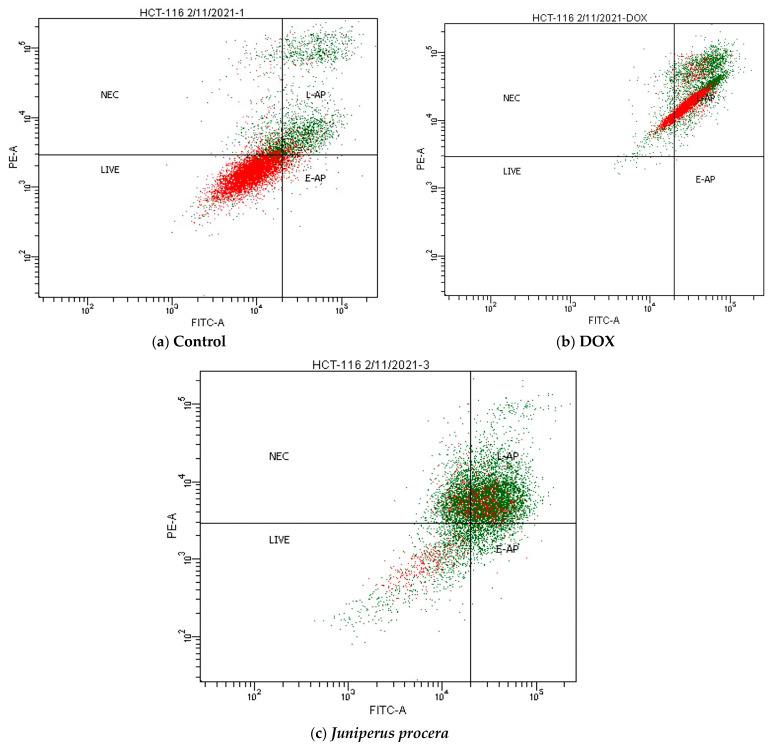
Flow-cytometry plots for HCT_116_ staining with Annexin V/7-PI: (**a**) control; (**b**) 3 μM DOX; (**c**) IC_50_ dosage of *J. procera* methanolic extract.

To evaluate the antiproliferative role of *J. procera* in human cancer cells, apoptosis and cell-cycle analysis were investigated in HCT_116_ cells treated with *J. procera* methanolic extract. As shown in Figure 5 and Table 4, the proportion of apoptosis and necrosis of HCT_116_ cells treated with *J. procera* methanolic extract in the upper and lower right quadrants of Figure 5 depicts late and early apoptotic cells, respectively. In the HCT_116_ cell line, the proportion of early and late apoptotic cells treated with *J. procera* methanolic extract was 3.8% and 44.8%, respectively. *J. procera* methanolic extract caused apoptosis and necrosis in 20% and 48.6% of HCT_116_ cells, respectively, compared to only 3.5% in untreated cells. Doxorubicin (DOX), a well-known chemotherapy drug, was used as a positive control.

**Figure 6:**
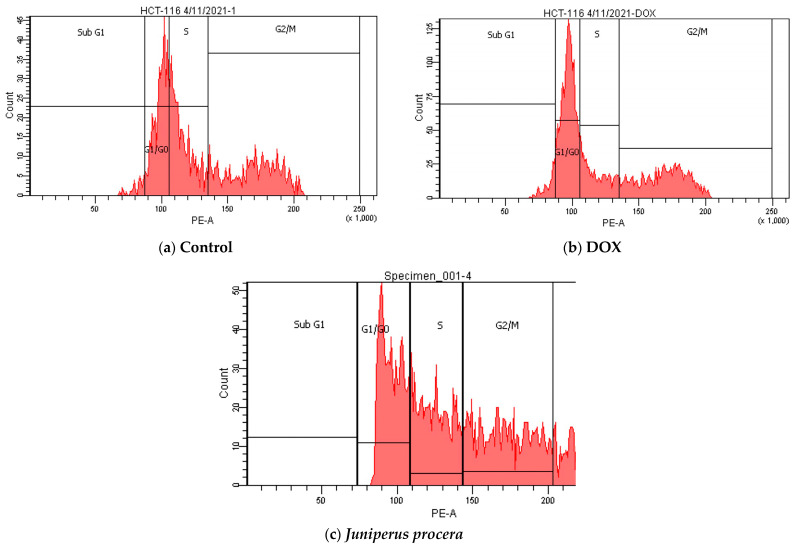
Cell-cycle arrest of untreated HCT_116_ (**a**), DOX (**b**), and *J. procera* methanolic extract (**c**).

**Figure 7:**
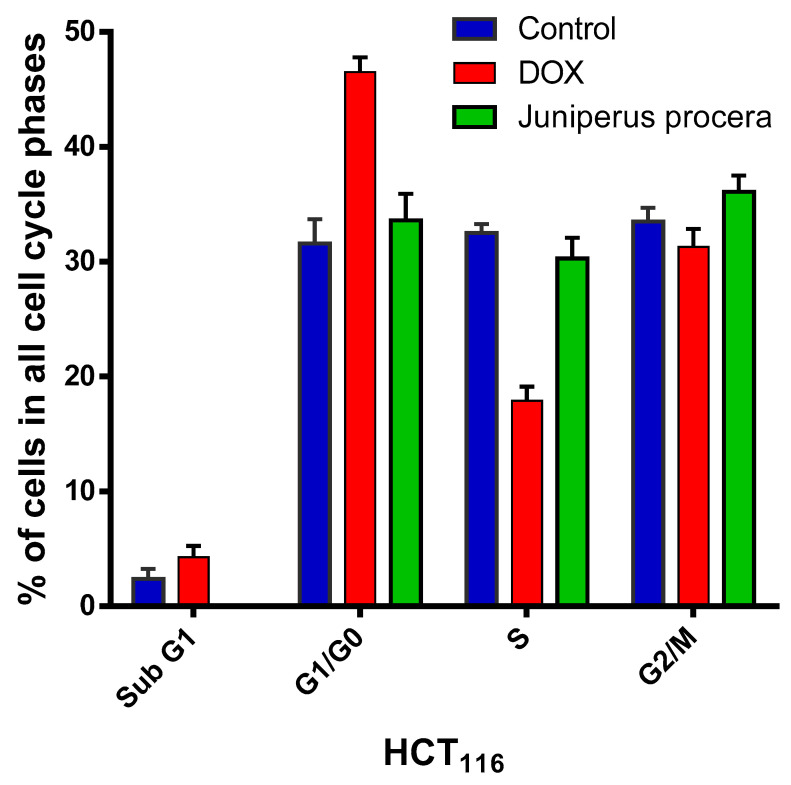
Percentage of cells of HCT_116_ treated with DOX and *J. procera* methanolic extract in all cell-cycle phases.

To further explore the notion that *J. procera* has metabolic activity and a viable role in HCT_116_ cells, a cell-cycle assay was performed. As shown in Figure 6 and Figure 7 and Table 5 and Table 6, the percentages of untreated HCT_116_ cells were 61.2%, 12.3%, and 25.8% in G1/G0, S, and G2/M, respectively. However, when treated with the IC_50_ of *J. procera* methanolic extract, HCT_116_ cells arrested in a lower proportion in the G1/G0 phase (33.6%), in a higher percentage in the S phase (30.3%), and in a slightly higher percentage in G2/M (36.1%).

The cell percentages in G1/G0 for DOX (an anticancer medication) and *J. procera* methanolic extract were 46.5 and 33.6, respectively. In the S phase, the percentage of cells treated with *J. procera* extracts was 30%, whereas the percentage of HCT_116_ cells treated with DOX was 17.9%. These results indicate that the percentages of HCT_116_ cells in S phases was more elevated in cells treated with *J. procera* than in those treated with DOX. Furthermore, in the G2/M phase, DOX (31.3%) and the extract had nearly identical effects (36.1%). Taken together, these data lead us to propose that *J. procera* may play an antiproliferative role in HCT_116_ cells via apoptosis and interruption of the cell-cycle process.

## 3. Discussion

The nature of the extraction solvents utilized is essentially what determines the yield of bioactive compounds, the type of compounds isolated, and the impact of biological activity In this investigation, *J. procera* extract was prepared using solvents of various polarities, including methanol, n-hexane, dichloromethane, and ethyl acetate. A previous study showed similar higher-yield results [13,22,23,24,25]. The largest concentration of extract in methanol solvent was associated with stronger polarity, and it is assumed that methanol may dissolve both hydrophilic and lipophilic elements in plants, resulting in a larger yield. Phytochemical analysis and cytotoxicity were carried out with a methanolic extract of *J. procera*.

The IC_50_ values were ranked as follows: JK-1 > HCT116 > MCF-7 > HepG2. The cytotoxicity of *J. procera* fruit and leaf extract has been compared in previous studies. Previous researchers found that *J. procera* fruit extract was more cytotoxic than *J. procera* leaf extract against breast (MCF-7 and MDA-MB-231) and ovarian (SKOV-3) cancer cells. However, the leaf extract was more cytotoxic to liver (HepG2) and cisplatin-resistant ovarian cancer cell lines (A2780CP) [20].

IC_50_ values of doxorubicin and a leaf extract of *J. procera* combined with doxorubicin in a treated ovarian cancer cell line (A2780CP) were almost similar, at 1.2 and 0.9 μg/mL. The methanolic extract of *J. procera* leaves demonstrated cytotoxicity on oral SCC-9 cell lines, with an IC_50_ value of 208.7 g/mL [13].

By combining silver nanoparticles with the *J. procera* extract, the cytotoxicity of the extract against the colon cancer (Caco2) cell line was improved [20]. The cytotoxicity of *J. procera* extract transformed into ZnO nanocomposites was also considerably increased [24]. Extracts from a different *Juniperus* species are believed to have anticancer properties. *Juniperus communis* has differing degrees of cancer-cell-proliferation inhibition that may be extracted using various solvents. *Juniperus phoenicea* extracts created with different solvents, such as *n*-hexane, chloroform, and methanol, demonstrated that cell-proliferation was suppressed in human lung (A549), breast (MCF-7), and liver (HepG2) cancer cells, with the MCF-7 cell line being the most sensitive, with IC_50_ values of 24.5 μg/mL [25].

Stankovic and colleagues found that the different species of *Teucrium* extract on the HCT116 cell line displayed increased cytotoxicity at higher doses after 72 h of exposure. At lower doses and with longer exposure times, the extract stimulates some proliferative effects in surviving cells [26]. These findings show that the cytotoxic impact is affected by various parameters, including the kind of solvent used for extraction, plant components utilized, cell lines tested, and the treatment period.

Figure 4 depicts the molecular docking of 2*H*-1-benzopyran-3-carbothiamide (Hit 1) with 3ig7 CDK-5 (receptor). The CDK-5/Hit 1 complex was found in the residues Leu83, Lys33, Asp145, and Val18. The Hit 1 compound (ligand) interacts with Lys33 and Asp145 in CDK-5 (receptor) through a hydrogen bond. Lys33 was identified as an H-donor to the -NH group, whereas Asp145 was an H-acceptor from the -NH2 group. A hydrophobic interaction between Val18 and the aromatic ring of the Hit 1 chemical was found. The Hit 1 chemical interacts with Leu83 through a backbone interaction via an electron-withdrawing nitro group. In the erythroid spectrin/Hit 1 (1woa/Hit 1) complex, the residues contribute the most to Ser52 and Tyr53. The interaction of Ser52 and Tyr53 with the aromatic ring of the Hit 1 chemical was recognized as a hydrophobic interaction.

The molecular docking approach is used to anticipate the probable orientation of the ligand and receptor that results in the formation of a stable complex [27]. The chemical in a plant extract is referred to as a ligand, while the protein in cancer cells is referred to as a receptor. The binding affinity of the ligand and receptor can be used to predict the affinity and activity of a therapeutic molecule. It is also essential for comprehending the anticancer processes via which the active substance in the plant leaves may be detected.

In this study, molecular docking was performed on 12 different molecules identified by GC/MS and proteins in cancer cells, such as cyclin-dependent kinase 5, cytochrome P450 aromatase, erythroid spectrin, and topoisomerase. We found that 2-imino-6-nitro-2*H*-1-benzopyran-3-carbothiamide docked with all four targeted receptors mentioned above.

Cdk5 has lately been implicated in the formation and progression of several malignancies, including colon tumors [24,28]. Cdk5 (3ig7) is a viable therapeutic target receptor for developing novel cancer medicines due to its extensive protumorigenic activity. Cytochrome P450 aromatase is an enzyme responsible for catalyzing the estrogen hormone, which is known as a proliferative factor in breast cancer. Based on our docking data, Hit 1 docks with cytochrome P450 aromatase, which may lead to a reduction in the oncogenic activity of estrogen [29]. The erythroid spectrin is known for its important role in maintaining cell-membrane integrity and its contribution to the cell cycle and cell spreading. Here, we have shown that the chemical Hit 1 docked with the erythroid spectrin, which may prevent its role in the proliferation and spreading of cancer cells [30]. Topoisomerase is a critical enzyme in DNA strand cleavage that acts as a cellular controller during replication and transcription. We found that the Hit 1 compound docked with topoisomerase, which may result in a reduction in cancer-cell proliferation [31].

Nitroaromatic compounds are thought to be prodrugs for cancer treatment [32]. Nitroaromatic groups are thought to be trigger units that can take up to six electrons from reductase enzymes. This results in the creation of different reduced species and radicals, with a significant shift in electron density at nitrogen-carrying substituents, which might increase cellular toxicity as they act as DNA crosslinking agents and undergo sequential inhibition in the cell cycle.

Flow cytometry was used to examine apoptosis and necrosis in colon cancer cells (HCT_116_). Apoptosis is a type of programmed cell death that may be detected via DNA damage. It is a valuable marker for selecting chemicals for further research as potential anticancer medicines. The cell releases phosphatidylserine on the extracellular surface during apoptosis, which may be detected using Annexin V-FITC/propidium iodide (PI) fluorescence. Annexin V is a protein linked to a fluorescent green dye that indicates apoptosis. Propidium iodide (PI) is a fluorescent red dye that stains necrotic and late-apoptotic DNA. Comparative research was conducted under three distinct settings, including a control (untreated), an IC_50_ dosage of doxorubicin (DOX), and *J. procera* methanolic extract, to evaluate the amount of cell apoptosis and necrosis in the colon cancer cell (HCT_116_). These findings suggest that *J. procera* leaf extract may efficiently trigger apoptosis and necrosis in HCT_116_ cells.

To determine the level of cell-cycle arrest caused by *J. procera* extract at a certain phase, the percentages of the cell population in the interphase (G0, G1, S, and G2) and mitotic phase (M) were measured using flow cytometry and propidium iodide (PI) labeling. HCT_116_ cells treated with *J. procera* methanolic extract halted an increase in the S and G2/M phases by 2.5 and 1.4 times, respectively, and they reduced levels by 1.8 fold in the G1/G0 phases.

DOX is less effective than *J. procera* extract at moving cells from the G1/G0 phase to the next phase of the cell cycle. In contrast to DOX, the extract increases the number of cells in S-phase *J. procera* extracts (30%). The number of cells in the G2/M phase increases significantly following JP extract treatment when compared to control and DOX. Furthermore, in the G2/M phase, DOX and the extract have nearly identical effects. It has been observed that anticancer medicines halt the cell cycle through a series of processes that occur at distinct stages in G1 or G2/M, followed by cell death via apoptosis [33]. During cell-cycle inhibition, anticancer drugs may cause DNA damage by causing cell stasis at various stages of the cell cycle, such as at G1 or G2/M, and thereby induce apoptosis. *J. procera* extracts have a cytotoxic impact on cancer cells via cell-cycle arrest, which is produced by DNA damage and the stalling of cells at the G1 or G2/M phase, resulting in apoptosis. As a result, *J. procera* methanolic extract has apoptosis-inducing properties. The response of the extract and reductions in cell growth depend on the cell line, the concentration of the extract, and the treatment time [18,34]. The proportion of cells in the cell-cycle phase was determined after 24 h in the current investigation. By prolonging the treatment duration, it is feasible to reach the level of the most arrested cells at various stages of the cell cycle. Collectively, our demonstration of the anticancer action of *J. procera* methanolic extract emphasizes the need for more studies on the bioactive compounds that may inhibit specific oncogenic targets in cancer cells.

## 4. Materials and Methods

### 4.1. Plant Material

The herbalist Mr. Ali Mdawei collected fresh *J. procera* Hochst ex Endl. (Cupressaceae) leaves in April 2021 from Bahat Rabia, Asir region, Saudi Arabia (GPS coordinates 18.326245 0N, 42.321546 0E). The leaves were washed under running water, dried in the shade, and then ground into powder.

### 4.2. Extraction of Plant Material

The solvent *n*-hexane, dichloromethane, ethyl acetate, or methanol was used to extract air-dried, milled *J. procera* leaves (50 g/500 mL) for 24 h in a Soxhlet extractor, yielding 1.5, 0.9, 1.1, and 2.2 g of extract, respectively. To obtain phenolic bioactive components, methanol *J. procera* extract was treated using the GC/MS spectroscopic method.

### 4.3. Human Cell Line and Culture Conditions

Four human cell lines were available from the Tissue Culture Unit, Department of Biochemistry, Faculty of Science, King Abdulaziz University: human colorectal carcinoma cell line (HCT_116_), hepatoma G2 (HepG2), breast cancer cell line (Michigan Cancer Foundation–7) (MCF-7), and hemopoietic erythroid cell line (JK-1). The attached human cell lines were grown for 24 h in complete media, Dulbecco’s Modified Eagle Medium (DMEM), and JK human cell lines in Roswell Park Memorial Institute medium (RPMI 1640, which contains 10% fetal bovine serum and 1% antibiotic). The DMEM and RPMI 1640 were supplied by Life Technologies Gibco. The cells were incubated in a 5% CO_2_ incubator at 37 °C and 95% humidity.

After receiving 4 mL of 0.25% trypsin with EDTA, 90% of the confluent cells were collected and incubated in a CO_2_ incubator for 5 min. After 5 mL of complete medium was added, the trypsin process was stopped. The media-containing unattached cells were centrifuged, and the pellets were washed twice with sterile phosphate-buffered saline (PBS) [35,36].

The number of cells was determined using a hemocytometer and counted in the four primary squares after 20 μL of this cell-containing media were stained with 0.4% trypan blue. The number of cells per ml was calculated using the following equation: 1/4 × 10^4^ × 2. A total of 0.1 mL of 5000 cells suspended in complete media was placed in each well of a 96-well microplate, and the plate was then incubated in the incubator for 24 h.

### 4.4. Evaluations of the IC_50_ of J. procera in Human Cell Lines Using the MTT Assay

Using the MTT assay to evaluate the metabolic activity and cell viability of cancer cells, *J. procera* methanolic extract’s potency as an anticancer agent was assessed. Different amounts of *J. procera* methanolic extract, ranging from 12.5 to 200 μg/mL, were applied to the media once 70% of the cells in each well had reached confluence. We repeated each concentration 4 times. Then, 96-well plates were incubated for 48 h before the media in each well were replaced with 100 μL of free media containing 0.5 mg of MTT/mL for 4 h in the incubator. Dimethylsulfoxide (DMSO) at a dosage of 100 μL was added to each well and left to remain at room temperature for 15 min before being detected at 595 nm with a microplate reader (Bio-RAD microplate reader, Hercules, CA, USA). Using the curve of cell viability vs. different concentrations of *J. procera* extract, the 50% inhibitory concentration (IC_50_) of *J. procera* methanolic extract against cell lines was calculated [35,36].

### 4.5. Assessment of Apoptosis in HCT_116_ Cells Treated with J. procera

For 24 h, HCT_116_ was grown in a CO_2_ incubator. Cells were divided and counted using trypsin. In a 6-well plate, 2 × 10^5^ cells were grown for 24 h. The well’s medium was changed to complete media containing the IC_50_ of the methanolic extract of *J. procera*. Trypsin was used to separate the HCT_116_ cells after 24 h, and the medium from each well containing cells was then gathered into tubes and centrifuged. After that, phosphate-buffered saline (PPS) solution was used to wash the pellets. To 100 μL of suspended treated HCT_116_ cells, 400 μL of binding buffer and 25 μL of Annexin V-FITC/propidium iodide (PI) solution were added. The cells were detected using a flow-cytometry device. The software module computed data automatically [37].

### 4.6. Evaluation of the Cell Cycle in HCT116 Treated with J. procera

PI from ThermoFisher Scientific can attach to DNA, stain it, and measure cellular aggregation throughout the cell cycle using flow cytometry [38]. A total of 1 × 10^6^ HCT_116_ cells were cultivated on a 6-well plate for 24 h. The medium was replaced with a medium containing *J. procera* extract at the IC_50_ level. To collect the treated HCT_116_ cells after 24 h, 0.5 mL of 0.25% trypsin was added to each well, and trypsin activity was stopped with 0.5 mL of complete medium. The suspended cells were rinsed twice with PBS after centrifuging for 5 min at 1500 rpm. The cells were placed in 1 mL of ice-cold 70% ethanol and frozen for at least 4 h at −20 °C. After a 100 μL wash with cold PBS containing RNase A, the suspended cells were stained with 250 μL of PI solution (50 mg/mL PI) and allowed to rest in the dark for 1 h. Every cell that had been designated was read using a flow cytometer (Applied Bio-system, Hercules, CA, USA).

### 4.7. GS/MS Examination of Methanolic Extract of J. procera Leaves

*J. procera* extract was phytochemically analyzed using a gas chromatograph (Agilent Technologies, Santa Clara, CA USA) with mass spectrometry (GC/MS 7890B). The GC/MS device has a 59,778 mass-selective detector and an HP-5MS 30 m GC column (30 m length, 0.25 mm inner diameter, and 0.25 m film thickness). Helium (99.99% purity) was used as the carrier gas, with a column flow rate of 1 mL/min. Agilent Technologies’ ChemStation program (Agilent Technologies 7890B) was utilized for system control and data processing. The splitless injection mode was used, with a 1 L injection volume and a split ratio of 1:10. The temperature of the input injection was fixed to 250 °C. The column temperature schedule was as follows: 50 °C for 1 min, 50 °C to 200 °C for 10 min and held at this temperature for 5 min, and 200 °C to 300 °C for 15 min and held at this temperature for 10 min, for a total run time of 37.6 min. Electron impact ionization (EI) at 70 eV was used to ionize the ions in mass spectrometry, and spectra were monitored in the selected ion monitoring (SIM) mode with an m/z ratio of 40 to 500 or a time-of-flight detector. The bioactive components’ chemical names, molecular formulas, and molecular structures were determined by comparing them to the spectrum of known components listed in the NIST library (National Institute of Standards and Technology) (NIST 2.0).

### 4.8. Molecular Docking Study of J. procera Extract with Four Different Cancer Proteins

The molecular interactions between the active substances extracted from *J. procera* that theoretically bind to four different active proteins impact cell proliferation, receptors, the integrity of cell membranes, and DNA conformation. Molecular docking was applied to investigate cyclin-dependent kinase 5 (Cdk5) in colon cancer (PDB code = 3ig7 [28], aromatase cytochrome P450 in breast cancer (PDB code = 3eqm) [29], the N-terminal domain of the erythroid spectrin in erythroid cancer (PDB code = 1owa) [30], and topoisomerase in liver cancer (PDB code = 4fm9)” [31]. The MOE 2019.102 platform was used for all docking research. Each bioactive compound and all protein interactions were simulated in 2D and 3D. The binding energies (E) and chemical interactions of the 12 drug-like compounds docked to the protein targets were thoroughly examined.

**Figure 8:**
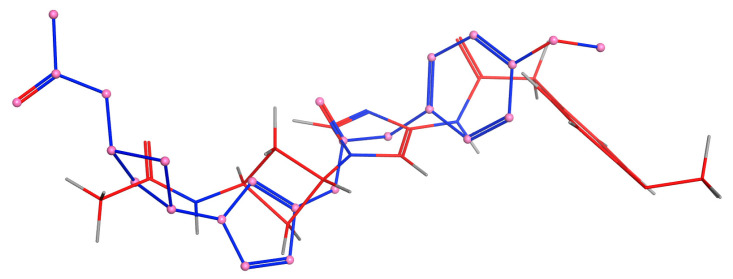
RMSD discrepancy between the docked molecule (in red) and the downloaded reference compound (in blue).

Protein 3D structures were retrieved as pdb files from the Protein Data Bank https://www.rcsb.org/ (accessed on 3 February 2023) after eliminating all solvent molecules and correcting all structures and charges, as previously reported [39]. Active sites were defined as the presence of the active medication or cocrystalline ligand and were isolated as dummy atoms. The docking results were generated by utilizing Triangle Matcher with stiff protein, and the docking score was determined for 30 postures using the London dG method, with the best 5 poses abstracted. The docking score (S), the root-mean-square deviation (RMSD) between the cocrystal and docked conformation, and the binding energies (Es) of each plant-derived molecule were used to calculate the findings. To determine the variations in binding affinities, the binding energies of each chemical were compared. The molecular interactions of the best-docked compounds with the target proteins were thoroughly investigated (Figure 8).

The cocrystalline molecule N-1-[*cis*-3-(acetylamino)cyclobutyl]-*1H*-imidazol-4-yl-2-(4-methoxyphenyl)acetamide was obtained from the database and used for the validation of the docking methods. The docked structure had a −7.86 kcal/mol docking score and an RMSD of 1.198.A^0^. The machine used for this investigation was configured with Windows 10 and an Intel (R) Core (TM) i7-8550U CPU running at 1.80 GHz and 1.99 GHz.

### 4.9. Statistical Analysis

The results are presented as the mean SDs of the viability of treated cells. The absorbance of treated cells * 100 divided by the absorbance of untreated cells was used to compute the percentage of viability. GraphPad Prism Software (version 9.0, San Diego, CA, USA) was used to determine the drug IC_50_. The flow-cytometry software from Applied Biosystems determined the percentage of cells in each phase, as well as the quantity of necrotic and apoptotic cells, automatically.

## 5. Conclusions

This study demonstrates that *J. procera* leaf extract is cytotoxic to the cancer cell lines HepG2, MCF-7, HCT116, and JK. Additionally, *J. procera* leaf extracts cause the death of HCT116 cells by arresting the cell cycle, which is caused by DNA damage and results in cell-stalling at the G1 or G2/M phase. A GC/MS analysis of *J. procera* leaf extracts showed 12 unique bioactive components. These findings imply that the extract of *J. procera* leaves contains bioactive substances that may be used as anticancer medications. A decrease in breast cancer cell-proliferation may also result from Hit 1 inhibition of aromatase, a cytochrome P450 enzyme that catalyzes estrogen generation in breast cancer patients with positive estrogenic receptors (MCF-7). Additionally, the function of erythroid spectrin in maintaining the integrity and adherence of cell membranes may be impacted by Hit 1’s reduction in production on the JK cell line. The results of molecular docking suggest that Hit 1’s reduction in proliferation and cell-cycle arrest in liver cancer cells may be caused by topoisomerase inactivation. Purification of the bioactive compound 2-Imino-6-nitro-2H-1-benzopyran-3-carbothiamide from *J. procera* is required to investigate its cytotoxicity against various cancer cell lines in vitro and in vivo.

## Figures and Tables

**Table 1:** The IC_50_ of *J. procera* methanolic extract in HCT_116_, HepG2, MCF-7, and JK after 48 h.

IC_50_ (μg/mL)	HCT_116_	HepG2	MCF-7	JK
**Range of IC_50_ (μg/mL)**				
	93–142	60–96	96–130	104–150
			
**Average Value of IC_50_ (μg/mL)**	115	75	112	124
			

**Table 2:** Identification of the bioactive components in *J. procera* methanol leaf extract using GC/MS.

No.	Compound Name	Molecular Formula	RT (Min)	RC (%) Rel. %
**1**	2-Imino-6-nitro-2*H*-1-benzopyran-3-carbothiamide	C_10_H_7_N_3_O_3_S	4.079	0.49
**2**	5-Methylpyrimidine-2,4-(1*H*,3*H*)-dione (Thymine)	C_5_H_6_N_2_O_2_	4.079	0.47
**3**	2,4-Dihydroxy-2,5-dimethyl-3(2*H*)-furan-3-one	C_6_H_8_O_4_	5.666	0.10
**4**	2,3-Dihydro-3,5-dihydroxy-6-methyl-[4*H*]-pyran-4-one	C_6_H_8_O_4_	8.09	0.40
**5**	5-Hydroxymethylfurfural	C_6_H_6_O_3_	9.051	1.24
**6**	2,3-Dihydrobenzofuran	C_8_H_8_O	9.223	0.12
**7**	3,7-Dimethyloct-6-en-1-ol	C_10_H_20_O	14.206	1.91
**8**	3-*O*-Methyl-*D*-glucose (3-Methylglucose)	C_7_H_14_O_6_	14.895	1.03
**9**	2-Methyl-1,3-dioxolane-2-propanal	C_7_H_12_O_3_	15.413	11.40
**10**	Tetrahydro-2-methylthiophene	C_5_H_10_S	15.601	2.00
**11**	2-Butyl-2-methyl-1,3-dioxolane	C_8_H_10_O_2_	16.253	5.94
**12**	1-Amino-1-cyclobutanecarboxylic acid	C_5_H_9_O_2_N	16.339	2.10

**Table 3:** Docking score (S) and root-mean-square deviation (RMSD) of all Hit compounds as ligand molecules against target proteins 3ig7, 1woa, 3eqm, and 4fm9.

Hit	Colon		Erythroid		Breast		Liver	
	3ig7		1owa		3eqm		4fm9	
	S	RMSD	S	RMSD	S	RMSD	S	RMSD
**Hit 1**	−5.56	0.81	−6.71	2.03	−5.88	1.31	−5.66	0.90
**Hit 2**	−4.28	1.75	−3.74	0.53	−4.47	0.53	−4.62	1.07
**Hit 3**	−4.51	0.69	−4.05	0.94	−5.07	3.52	−5.02	1.65
**Hit 4**	−4.50	0.65	−3.99	0.72	−4.76	2.89	−4.99	1.55
**Hit 5**	−4.22	0.79	−3.91	1.76	−4.67	2.09	−4.55	1.19
**Hit 6**	−4.43	1.43	−4.03	1.11	−4.53	1.10	−4.69	0.91
**Hit 7**	−5.34	1.28	−4.39	2.00	−5.69	0.82	−5.43	0.80
**Hit 8**	−5.07	1.11	−4.23	1.55	−5.45	0.96	−5.28	2.16
**Hit 9**	−4.86	2.09	−4.20	1.53	−5.10	0.98	−5.07	1.04
**Hit 10**	−4.15	2.66	−3.70	0.94	−4.38	2.56	−4.13	1.76
**Hit 11**	−5.17	0.58	−4.31	0.81	−5.43	1.66	−5.33	1.23
**Hit 12**	−4.22	1.15	−3.56	3.80	−4.45	4.00	−4.29	0.95

**Table 4:** Docking interactions and energies of 2*H*-1-benzopyran-3-carbothiamide with the receptors cyclin-dependent kinase 5 (3ig7), erythroid spectrin (1woa), aromatase cytochrome P450 (3eqm), and topoisomerase (4fm9).

	Ligand	Receptor	Interaction	Distance	E (kcal/mol)
**3ig7**	N 16	OD1 ASP 145(A)	H-donor	2.85	−2.0
	O 21	N LEU 83 (A)	H-acceptor	3.03	−1.3
	N 23	NZ LYS 33 (A)	H-acceptor	3.02	−9.7
	6-ring	CG2 VAL 18 (A)	pi-H	4.40	−0.5
**1woa**	6-ring	CA SER 52 (A)	pi-H	3.59	−1.1
	6-ring	N TYR 53 (A)	pi-H	4.43	−1.2
**3eqm**	S 19	CA VAL 373	H-acceptor	3.68	−1.1
	S 19	N MET 374	H-acceptor	3.28	−2.7
**4fm9**	S 19	NE ARG 673 (A)	H-acceptor	2.98	−2.5
	S 19	NH1 ARG 673 (A)	H-acceptor	2.36	−1.8

**Table 5:** Early and late apoptotic percentages in HCT_116_ cells treated for 24 h with 3 μM DOX and *J. procera* extract.

Treatment	% of Viable Cells(Lower Left)	% of Necrosis(Upper Left)	% of Early Apoptosis(Lower Right)	% of Late Apoptosis(Upper Right)	Total % of Apoptosis
**Control**	93.7	3.2	1.1	2	3.2
**DOX 3**	0	10.7	0	89.3	89.3
***J. procera***	31	20	3.8	44.8	48.6

**Table 6:** Percentage of HCT_116_ cells treated with DOX and the IC_50_ of *J. procera* methanolic extract for 24 h.

	Drugs	Control	DOX	*J. procera* Extract
Phase				
**Sub G1**		2.4	4.3	0
**G1/G0**		31.6	46.5	33.6
**S**		32.5	17.9	30.3
**G2/M**		33.5	31.3	36.1

## Data Availability

The data presented in this study are available upon request from the authors.

## Sample Availability

Samples of the compounds are available from the authors.
